# Anorexia nervosa restricting type has increased in severity over three decades: Japanese clinical samples from 1988 to 2018

**DOI:** 10.1002/eat.23418

**Published:** 2020-11-27

**Authors:** Tomoko Harada, Tsuneo Yamauchi, Dai Miyawaki, Saori Miyamoto, Hisako Yoshida, Kazuya Nishimoto, Takumi Matsuzuka, Mihoko Honda, Koki Inoue

**Affiliations:** ^1^ Department of Neuropsychiatry Osaka City University Graduate School of Medicine Osaka Japan; ^2^ Department of Medical Statics Osaka City University Graduate School of Medicine Osaka Japan

**Keywords:** anorexia nervosa, Japan, restricting type, time trend

## Abstract

**Objective:**

Although eating disorders (EDs) surged in the late 1900s and are now recognized worldwide, the time trend of ED characteristics remains unknown. This study aimed to clarify changes in characteristics of anorexia nervosa restricting type (AN‐R) over 30 years.

**Methods:**

We conducted a cross‐sectional study and examined 996 female treatment‐seeking patients with AN‐R in Japan from 1988 to 2018. Demographics, body mass index (BMI), and Eating Disorder Inventory scores were compared among three groups in accordance with the time of initial consultation: Group 1 (1988–1998), Group 2 (1998–2008), Group 3 (2008–2018).

**Results:**

The mean BMI at the initial consultation significantly decreased by 0.6 kg/m^2^ (from 14.0 kg/m^2^ in Group 1 to 13.4 kg/m^2^ in Group 3). Groups 2 and 3 scored significantly higher in drive for thinness, interpersonal distrust, and interoceptive awareness than those in Group 1. The range of onset age is wider and the number of late‐onset AN‐R with prolonged delay in treatment has increased over time.

**Discussion:**

This study shows that AN‐R has increased in physical and psychopathological severity over the past 30 years in Japan. Interdisciplinary research is needed to clarify the relationship between AN‐R and time trend.

## 1 INTRODUCTION

Eating disorders (EDs) are serious psychiatric disorders characterized by abnormal eating or weight‐control behaviors that most likely occur in females than males. The conceptualization and manifestation of EDs have changed over time. In the late 17th century, Morton (1694) first described EDs as a medical condition. The term *anorexia nervosa* (AN) was first used to describe a self‐starvation syndrome by Gull (1873). Afterward, EDs became apparent in many parts of the world in the latter half of the 20th century (Pike & Borovoy, 2004).

In Japan, some case reports of AN were seen in the 1960s (Ishikawa, Iwata, & Hirano, 1960), while an increasing number of cases of AN and bulimia nervosa (BN) were reported in the 1970s (Pike & Borovoy, 2004). Correspondingly, a cross‐sectional prevalence survey was started in Japan in 1981 (Nakai et al., 2018), which reported that the numbers of both AN and BN patients appeared to increase sharply in the 1980s. Nakai, Nin, and Noma (2014) said that the prevalence of EDs in Japan reached high levels in the 1980s, 10 years after the West, in comparison with Hoek's review, which presented the incidence of AN and BN to be around eight per 100,000 persons per year and 12 per 100,000 persons per year, respectively (Hoek, 2006; Japan Society for Eating Disorders, 2012). It is theorized that the increase in number of patients with EDs in the 1980s is greatly influenced by westernization, modernization, and urbanization (Pike & Borovoy, 2004).

EDs are influenced by sociocultural factors (Pike & Dunne, 2015). Although culture has changed over time, it is not clear how the pathological and behavioral characteristics of EDs have changed. Among EDs, AN restricting type (AN‐R) is the first described subtype and has the greatest diagnostic stability (Fichter & Quadflieg, 2007). Given the fact that its diagnostic criteria have hardly changed to date, we focused on AN‐R. To the best of our knowledge, there are no studies that report physical severity and ED‐related symptoms of AN‐R using a standardized measure over time. The purpose of this study is to clarify the changes in physical severity and ED‐related symptoms in AN‐R over time.

## 2 METHODS

### 2.1 Participants

We conducted a cross‐sectional study based on the medical records of ED treatment‐seeking patients who consulted the Department of Neuropsychiatry at Osaka City University Hospital during three decades—from April 1988 to March 2018. This facility is a 980‐bed hospital that provides both primary care and highly advanced medical treatment to the local community of Osaka city, an urban area in Japan. Patients with EDs have consulted the facility without age and economical restrictions because of the Japanese universal healthcare insurance, thus avoiding referral bias.

At the initial consultation, we collected data that contained BMI history up to that point as well as ED‐related symptoms.

Patients were diagnosed by experienced psychiatrists in EDs based on the diagnostic criteria of EDs at the time of consultation—the Diagnostic and Statistical Manual of Mental Disorders (DSM) third edition (American Psychiatric Association, 1980), revised DSM third edition (American Psychiatric Association, 1987), DSM fourth edition (American Psychiatric Association, 1994), and revised DSM fourth edition text revision (American Psychiatric Association, 2000)—but were subsequently rediagnosed based on their medical records using the new diagnostic criteria, DSM fifth edition (DSM‐5) (American Psychiatric Association, 2013).

The inclusion criteria were as follows: (a) female, (b) diagnosed with AN‐R according to the guideline of DSM‐5, and (c) Japanese speaker.

### 2.2 Procedures

The participants were divided into three groups (per decade) based on their initial consultation date to the unit (Group 1: April 1988 to March 1998; Group 2: April 1998 to March 2008; Group 3: April 2008 to March 2018). Patient background, such as current age, age of onset, duration of illness, delay in treatment of EDs—which refers to the duration from the onset of AN to the first medical treatment, body mass index (BMI) at the initial consultation, premorbid BMI, minimum BMI, and maximum BMI, were compared among the three groups.

Eating Disorder Inventory (EDI) (Garner, Olmstead, & Polivy, 1983) subscale scores were compared among the three groups, if available. Regarding delay in treatment, we additionally compared participants aged 30 years or younger only. This research was approved by the Ethics Committee of the Osaka City University.

### 2.3 Measure

The EDI is a widely used multidimensional inventory, consisting of a 64‐item self‐report questionnaire measuring psychological and behavioral traits common among patients with EDs. The EDI consists of a total of eight subscales, including three subscales that assess specific attitudes and behaviors concerning eating, weight, and body shape (drive for thinness, bulimia, and body dissatisfaction), and five subscales that assess psychopathologies relevant to EDs (ineffectiveness, perfectionism, interpersonal distrust, interoceptive awareness, and maturity fears). The validity of the Japanese versions of these measures has been previously shown (Shimura, Horie, Kumano, Sakano, & Suematsu, 2003). The EDI subscale scores for Japanese healthy participants have been shown to be as follows: age: 18.7 ± 0.6 years, BMI: 19.7 ± 1.9 kg/m^2^, drive for thinness: 6.7 ± 5.3, bulimia: 2.5 ± 3.8, body dissatisfaction: 14.9 ± 7.1, ineffectiveness: 6.4 ± 5.7, perfectionism: 3.3 ± 3.4, interpersonal distrust: 3.2 ± 2.8, interoceptive awareness: 3.5 ± 4.7, and maturity fears: 6.5 ± 4.0 (Kusano‐Schwarz & von Wietersheim, 2005).

### 2.4 Statistical analyses

Descriptive statistics were used to present the sample profile using means, *SDs*, and percentages. A series of analysis of variance (ANOVA) models were used to compare the three groups. Tukey's post hoc pairwise comparisons were used to examine significant group differences. To compare EDI subscales, analysis of covariance (ANCOVA) was used with age, duration of illness, and BMI as covariates. Two‐tailed *p* values less than .05 were considered significant. All analyses were performed using SPSS 23 for Mac OS X.

## 3 RESULTS

A total of 996 participants with AN‐R met the inclusion criteria. Of those, EDI data were available for 585 participants.

Of the 996 participants, the mean age at evaluation was 23.0 ± 8.7 years, mean age at ED onset was 19.5 ± 6.5 years, mean duration of illness was 3.6 ± 5.5 years, mean delay in treatment was 2.1 ± 3.9 years, mean current BMI was 13.6 ± 1.9 kg/m^2^, mean premorbid BMI was 19.1 ± 2.5 kg/m^2^, mean minimum BMI was 13.1 ± 1.9 kg/m^2^, and mean maximum BMI was 20.4 ± 2.9 kg/m^2^. Table 1 shows the demographic characteristics and BMIs among the three groups. Delay in treatment of Group 1 was significantly shorter than that of Groups 2 and 3. However, delay in treatment for participants under the age of 30 did not differ between groups (i.e., the mean ± *SD* is 1.0 ± 1.3, 1.4 ± 1.9, and 1.2 ± 1.8 years in Groups 1, 2, and 3, respectively).

**TABLE 1:** Time trend of demographics, body mass indices, and Eating Disorder Inventory subscales

	Group 1		Group 2		Group 3					
Demographics, body mass indexes (BMIs) (ANOVA)	*n* = 180		*n* = 467		*n* = 349					
	Mean	*SD*	Mean	*SD*	Mean	*SD*	*F*	*p* Value	*η* ^2^	Pairwise comparison
Age (years)	20.3	5.1	22.5	7.8	25.1	10.6	20.144	<.001	0.039	Group 1 < Group 2 < Group 3
Onset age (years)	18.3	4.0	19.1	5.8	20.7	8.0	10.523	<.001	0.021	Group 1, Group 2 < Group 3
Duration (years)	2.0	2.8	3.7	5.6	4.3	6.3	10.560	<.001	0.021	Group 1 < Group 2, Group 3
Delay in treatment (years)	1.2	1.9	2.1	3.5	2.7	5.0	7.416	.001	0.017	Group 1 < Group 2, Group 3
BMI (kg/m^2^)	14.0	1.8	13.7	1.9	13.4	1.9	5.860	.003	.012	Group 1 > Group 3
Premorbid BMI (kg/m^2^)	19.6	2.5	19.2	2.4	18.7	2.5	7.241	.001	.016	Group 1, Group 2 > Group 3
Minimum BMI (kg/m^2^)	13.4	1.9	13.1	1.9	12.9	1.9	4.376	.013	.009	Group 1 > Group 3
Maximum BMI (kg/m^2^)	20.4	2.6	20.5	3.0	20.3	3.0	0.296	.744	.001	

EDI subscales (ANCOVA)	Group 1		Group 2		Group 3					
	*n* = 115		*n* = 244		*n* = 226					
	Mean	*SD*	Mean	*SD*	Mean	*SD*	*F*	*p* Value	ηp^2^	Pairwise comparison
Drive for thinness	5.4	5.8	8.5	7.6	8.0	6.7	11.340	<.001	0.038	Group 1 < Group 2, Group 3
Bulimia	0.7	2.0	1.7	3.5	1.3	3.3	3.976	.019	0.014	Group 1 < Group 2
Body dissatisfaction	11.0	3.8	10.2	5.1	10.9	5.6	1.253	.287	0.004	
Ineffectiveness	9.7	6.0	10.5	6.6	11.8	7.4	1.962	.141	0.007	
Perfectionism	3.4	3.3	4.5	4.0	4.3	4.0	3.432	.033	0.012	Group 1 < Group 2
Interpersonal distrust	4.8	4.5	6.1	4.8	6.9	4.8	6.379	.002	0.022	Group 1 < Group 2, Group 3
Interoceptive awareness	6.9	6.2	9.5	7.9	9.5	7.7	7.015	.001	0.024	Group 1 < Group 2, Group 3
Maturity fears	6.8	4.4	7.2	4.3	7.8	5.3	2.510	.082	0.009	

*Note*: ANCOVA controls for age, duration, and BMI.

Abbreviations: ANCOVA, analysis of covariance; ANOVA, analysis of variance; BMI, body mass index; EDI, Eating Disorder Inventory; SD, standard deviation.

There were no significant differences in the main demographics (age, onset age, and BMI) between participants with and without EDI. Of the 585 participants, drive for thinness was 7.5 ± 6.9, bulimia was 1.3 ± 3.1, body dissatisfaction was 10.8 ± 5.1, ineffectiveness was 10.9 ± 7.0, perfectionism was 4.2 ± 3.9, interpersonal distrust was 6.2 ± 4.8, interoceptive awareness was 8.7 ± 7.5, and maturity fears was 7.4 ± 4.7. The EDI subscales of the three groups are also shown in Table 1.

**FIGURE 1:**
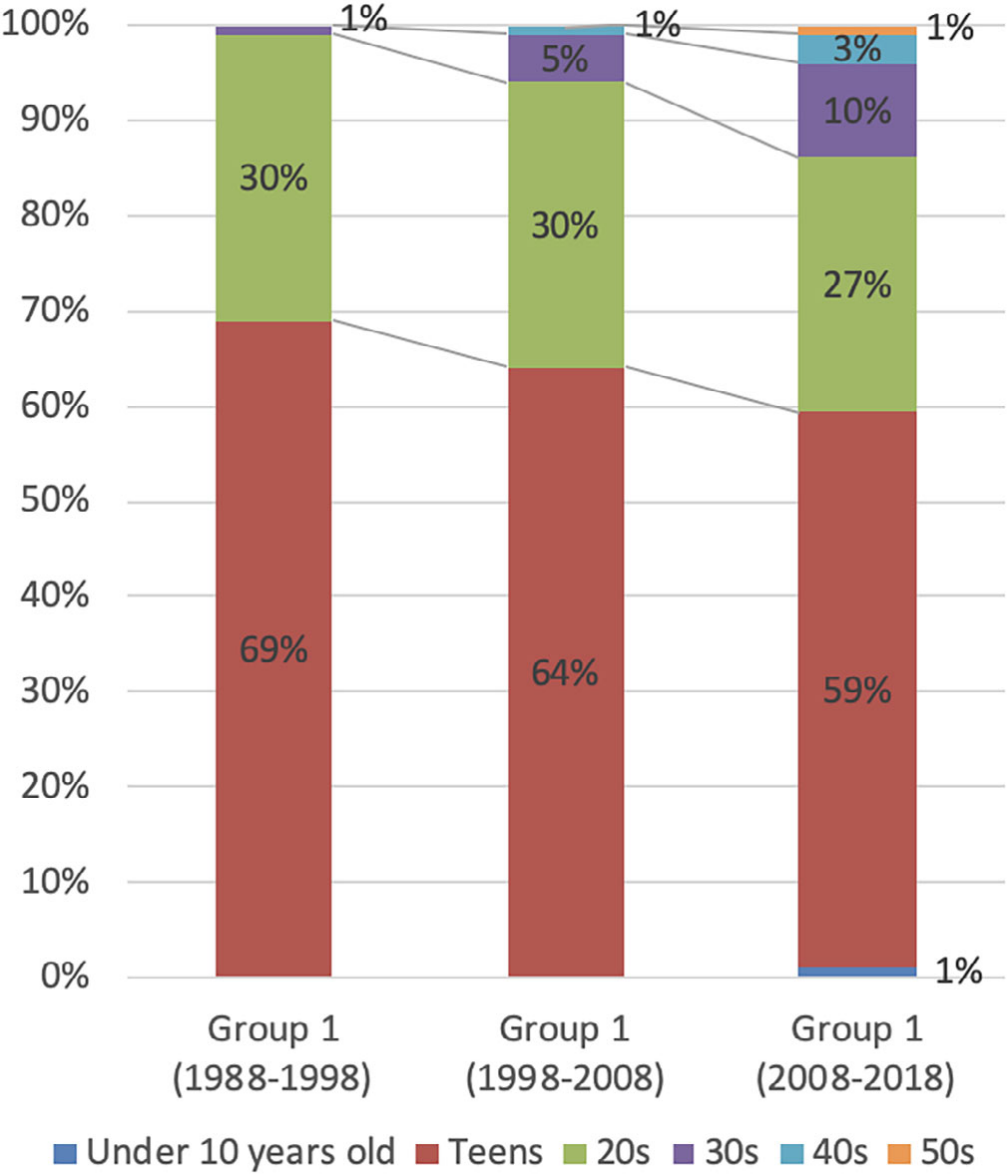
The proportion trend of the onset age for anorexia nervosa restricting type among the three groups. The information regarding onset age was collected at the initial consultation [Color figure can be viewed at wileyonlinelibrary.com]

Figure 1 shows the proportion trend of the onset age among the three groups.

## 4 DISCUSSION

We found that AN‐R has increased in physical and psychopathological severity for the past 30 years in Japan. To the best of our knowledge, this is the first study to clarify the time trend of severity in a large number of treatment‐seeking AN‐R patients.

The mean BMI of patients with AN‐R has decreased by 0.6 kg/m^2^ (from 14.0 to 13.4 kg/m^2^) over the past 30 years. This means that AN‐R has become physically severe over time in Japan. Since the DSM‐5 defines BMI <15 kg/m^2^ as the most severe, and Gaudiani et al. (2016) showed various physical risks with % ideal body weight <70% (i.e., BMI <15 kg/m^2^ in Japanese), the majority of treatment‐seeking female patients with AN‐R is considered severe. BMI cannot be simply compared with the results of Western countries due to differences in their physique, but also because of ethnic differences regarding BMI; for instance, the BMI of Asians is low, especially that of Japanese women (United States: BMI 29.2 kg/m^2^, China: BMI 23.7 kg/m^2^, Japan: BMI 22.1 kg/m^2^; World Health Organization, 2016). Meanwhile, a study in Japanese reported that the cut‐off value for refeeding hypophosphatemia was BMI < 12.6 kg/m^2^ (Yamazaki, Inada, & Yoshiuchi, 2019). Despite the small number of BMI decline, the time trend of a progressive decrease in BMI among Japanese patients with AN‐R is a serious problem to intervene.

This study showed that drive for thinness, which is the most central pathology of AN‐R, as well as interpersonal distrust and interoceptive awareness, is worse recently than in the past. Meanwhile, ineffectiveness, perfectionism, and maturity fears are the same.

The present study shows that the mean age of onset is higher and that the range of onset age is wider in the past 10 years. This is possibly related to the decline of onset age among patients with AN (Steinhausen & Jensen, 2015) as well as the increased number of late‐onset AN.

The period from the onset to the start of treatment has been longer over time. The present study shows that delay in treatment has gradually increased over the last 30 years (from 1.2 to 2.7 years). However, this interpretation must be used with caution, because the number of late‐onset AN with prolonged delay in treatment has increased over time. In fact, there was no group difference in delay in treatment for patients aged 30 years or younger.

The strength of this paper is that it examined the data of a large sample for 30 years, and adds to the non‐Western data, which has few reports. However, this paper has some limitations. First, this study used a retrospective design that relied on a self‐reported EDI questionnaire. Second, the data came from treatment‐seeking subjects visiting one unit only, resulting in the limitation regarding generalization of these findings. Third, the number of participants is different among the three groups. Japanese patients with EDs had increased in the 1980s–1990s, and had leveled off in the 2000s (National Center of Neurology and Psychiatry, 2020). Stability of AN incident rates in the 2000s was also reported in Western countries (Micali et al., 2017; Smink, van Hoeken, & Hoek, 2012). Thus, the difference in number of participants among the groups may reflect the time trend of the increase of patients with AN‐R, rather than the referral bias.

## 5 CONCLUSION

We found that the BMI and psychopathology of AN‐R tend to get worse as times change, particularly in recent years. In the future, interdisciplinary research is needed to examine the relationship between AN‐R and culture.

## CONFLICT OF INTEREST

The authors declare no potential conflict of interest.

## Data Availability

The data that support the findings of this study are available upon reasonable request from the corresponding author. The data are not publicly available due to privacy or ethical restrictions.
